# Genome-wide inference of the *Camponotus floridanus* protein-protein interaction network using homologous mapping and interacting domain profile pairs

**DOI:** 10.1038/s41598-020-59344-1

**Published:** 2020-02-11

**Authors:** Shishir K. Gupta, Mugdha Srivastava, Özge Osmanoglu, Thomas Dandekar

**Affiliations:** 1 Functional Genomics and Systems Biology Group, Department of Bioinformatics, Biocenter, Am Hubland, D-97074 Würzburg, Germany; 2 Department of Microbiology, Biocenter, Am Hubland, D-97074 Würzburg, Germany; 3 EMBL Heidelberg, BioComputing Unit, Meyerhofstraße 1, 69117 Heidelberg, Germany

**Keywords:** High-throughput screening, Cellular signalling networks

## Abstract

Apart from some model organisms, the interactome of most organisms is largely unidentified. High-throughput experimental techniques to determine protein-protein interactions (PPIs) are resource intensive and highly susceptible to noise. Computational methods of PPI determination can accelerate biological discovery by identifying the most promising interacting pairs of proteins and by assessing the reliability of identified PPIs. Here we present a first in-depth study describing a global view of the ant *Camponotus floridanus* interactome. Although several ant genomes have been sequenced in the last eight years, studies exploring and investigating PPIs in ants are lacking. Our study attempts to fill this gap and the presented interactome will also serve as a template for determining PPIs in other ants in future. Our *C. floridanus* interactome covers 51,866 non-redundant PPIs among 6,274 proteins, including 20,544 interactions supported by domain-domain interactions (DDIs), 13,640 interactions supported by DDIs and subcellular localization, and 10,834 high confidence interactions mediated by 3,289 proteins. These interactions involve and cover 30.6% of the entire *C. floridanus* proteome.

## Introduction

In terms of biodiversity and biomass, insects are the most successful animals on earth. They provide major beneficial impacts such as pollination, food source and soil improvement. On the contrary, some insects damage crops and spread deadly diseases as vectors. One of the harmful pests is the ant *Camponotus floridanus* which is widely distributed throughout Florida and the neighboring states^1^. They hollow the wood softened by moisture and damage the structural integrity of houses by affecting the wood work with their strong mandibles. Besides this, this ant species serves as a good model system to understand host-endosymbiont relationships regarding its bacterial endosymbiont *Blochmannia*^2^.

The complete genome sequences of *C. floridanus* has revealed the composition of proteins, based mainly on theoretical predictions utilizing their corresponding DNA sequence. We analyzed the transcriptome level evidence of protein existence and re-annotated the *C. floridanus* gene models and proteins^3^. How these proteins interact is not yet explored, in part due to limited genetic studies in this organism, high cost, time-consuming and labor-intensive nature of experimental methods. Protein interactions are at the core of nearly all biological processes, and knowledge about protein-protein interactions (PPIs) is vital for understanding biological systems. Despite advances in high-throughput experimental methods for detecting PPIs, the interaction networks for even the well-studied experimental model organisms are far from complete^4^. Nevertheless, high throughput assays typically include false positives PPIs^5^ which stipulate an enduring need for efficient computational methods to complement existing experimental approaches. In this context, combining the interolog method^6^ with adding domain information^7^, gene ontology (GO) annotation^8^ and cellular localization^9,10^ yields a graphical representation of the interaction networks, a robust and well-established approach to provide an intuitive vision and useful insights to help and analyze complex relations therein, as indicated by several previous studies in the reconstruction and understanding of PPIs in various organisms^11–14^. Here we used domain information, subcellular localization and isoform information to filter the preliminary global PPI network of *C. floridanus* reconstructed on stringent interolog based criteria. We focus on interactions predicted with high confidence to reduce noise. This conservative approach rejects 79.1% of the preliminary predicted interactions. We then explored the topologically important and evolutionary conserved proteins by analyzing the reconstructed interactome regarding cellular functions.

## Results and Discussion

### Generating the interactome of ant *C. floridanus*

PPIs are typically mediated by interactions between domains that are often evolutionary conserved across species^15^ and form stable interactions^16,17^. PPI (protein-protein interaction) maps from experiments on *D. melanogaster* were collected and augmented by PPI data from the DIP database (Database of Interacting Proteins). This provided a basis for interaction predictions according to interologs from *C. floridanus*: conserved proteins compared to Drosophila should also be conserved in their interactions^6,18^ (see Materials and methods for details).

**Figure 1:**
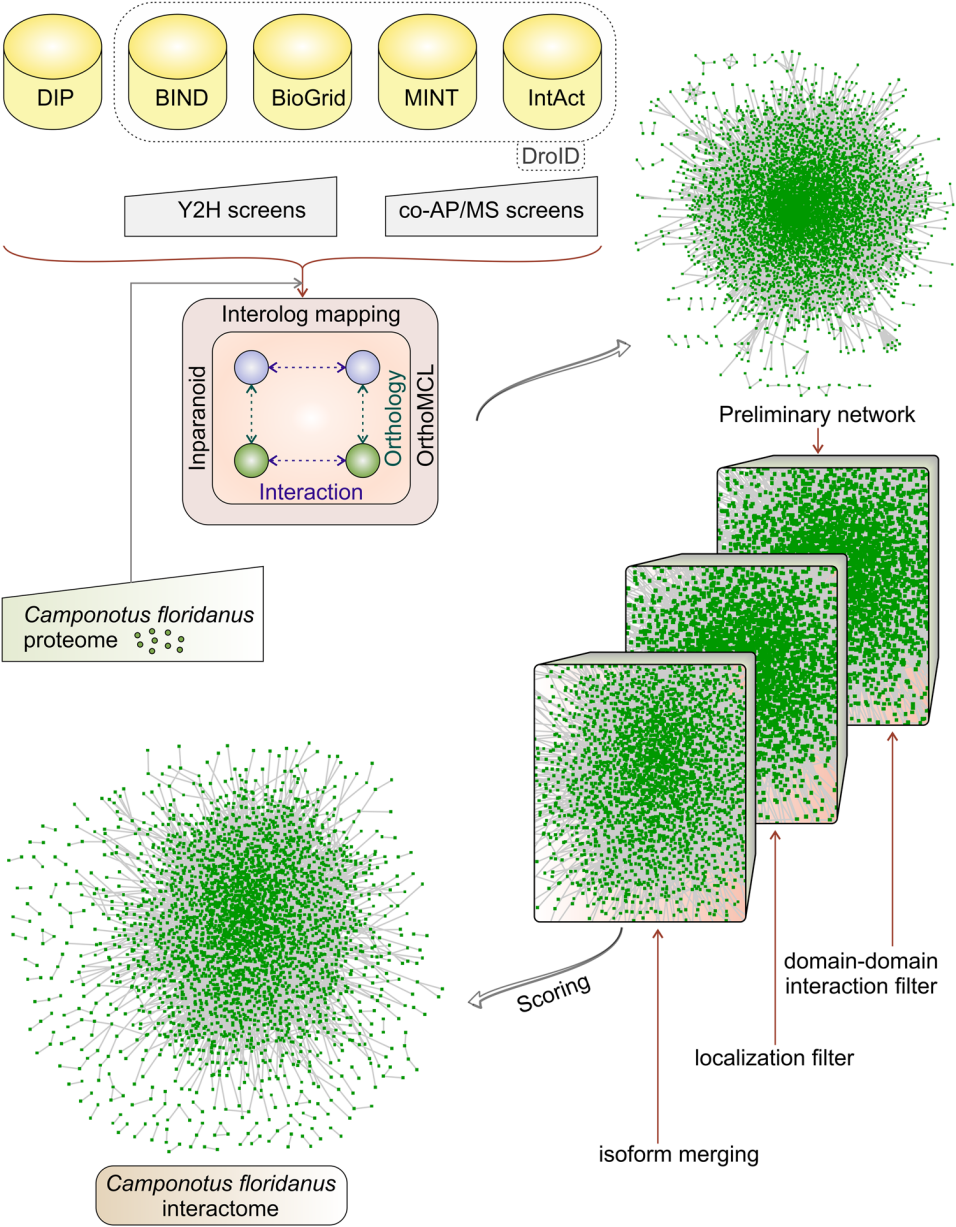
Pipeline for reconstruction of *C. floridanus* interactome. Top: Five databases, yeast two hybrid screens and coAP/MS screens and the *C. floridanus* interactome provide data for calculating conserved proteins (interologs) and protein-protein interactions. The preliminary protein-protein interaction network (top, right) is then filtered (right) by demanding in addition support by domain-domain interactions and shared subcellular localizations (right, bottom). It is then simplified merging similar isoforms (bottom, left).

Optimally, for such predictions several methods are combined^19^ (Fig. 1). We combined the orthology prediction methods InParanoid^20^ and OrthoMCL^21^. This did yield a first estimate of the *C. floridanus* interactome with 6274 nodes and 51866 edges^22^. However, the preliminary ant PPI network could have several false positive interactions acquired from the interologs of template data as shown previously in similar other studies^5,10,23,24^, including transfer to curated databases^25^. To reduce false predictions, we counter-checked all our data by domain-domain interactions (DDI). DDI are often used as an approach independent from sequence homology-based methods to predict protein-protein interaction networks and thus strongly reduce the number of false positives^7,26,27^. Generally, some of the PPIs are achieved via interactions between short motifs that are often transient interactions^28^. On the other hand, conserved interactions are mediated by conserved interaction domains across species^6^. Moreover, many signals and processes in the cell rely on conserved interacting protein domains^16,29^. There were 51866 conserved proteins (interologs) and 20544 ant protein-protein interactions that also were associated with DDI pairs, yielding a curated *C. floridanus* interactome with 4589 nodes and 20544 edges. For final curation of the interactome we used the subcellular localization of ant proteins: interacting proteins have to share the same subcellular localization (summarized in Table 1), predicted interactions between proteins not in the same location were removed. This led to a consolidated ant interactome consisting of 3914 nodes and 13640 edges. The highest proportion of interactions were identified in the cytoplasm followed by nucleus and plasma membrane respectively. A closer inspection of the interactions that were enriched across subcellular compartments (such as Golgi apparatus-cytoplasm) showed that in numerous cases at least one of the interacting proteins was alternatively localized to a compartment other than its major site of localization and thus the interacting proteins did indeed share a common compartment. For instance, in 482 interaction pairs (Table 1) at least one protein showed both the Golgi apparatus localization and cytoplasmic localization. It should be noted that these interaction partners are multiple localized proteins and may also appear in other cellular compartments. This is not an uncommon situation, as > 50% of proteins of our final interactome network annotated with predicted subcellular localization information are, in fact, localized at two or more compartments.

**Table 1:** Numbers of PPIs, by subcellular localization of interacting proteins in localization supported network.

Cytoplasm (2176)	6871										
Golgi apparatus (243)	482	720									
Peroxisome (21)	11	3	4								
Other (1) ^†^	1	0	0	1							
Nucleus (1866)	4686	227	3	0	6596						
Cytoskeleton (427)	1580	134	0	1	926	387					
Lysosome (50)	106	17	0	0	53	26	11				
Mitochondrion (319)	443	39	8	0	297	61	12	211			
ER (270)	368	59	0	1	179	79	19	24	155		
Extracellular (123)	120	16	4	0	87	36	1	19	22	51	
Plasma membrane (1522)	2365	383	10	1	1187	507	79	245	416	83	2636
	Cytoplasm (2176)	Golgi apparatus (243)	Peroxisome (21)	Other (1) ^†^	Nucleus (1866)	Cytoskeleton (427)	Lysosome (50)	Mitochondrion (319)	ER (270)	Extracellular (123)	Plasma membrane (1522)

The numbers in the parentheses indicate the total number of proteins in the interactome that are present in each subcellular compartment (based on localization prediction, including multiple localized proteins). ER, endoplasmic reticulum. ^†^The number given here in parentheses is the number of proteins that do not have subcellular localization in any of the other compartmenst in the table.

As a final step of network reduction, isoforms of proteins are shown as a single node. These steps of successive filtering ultimately reduce the complexity of the network and increase the confidence of the *C. floridanus* interactome. Figure 1 summarizes the *C. floridanus* protein-protein interaction databases, our workflow, pruning steps and resulting ant network. It consists of 3289 nodes and 10834 edges (more details in^22^). The complete four networks are provided in the Datasheets 1–4 in Supplementary Material. We also identified several novel interactions predicted to be present in *C. floridanus*. For instance, an interaction was observed between S-phase kinase-associated protein 1 (SkpA, Cflo_N_g10272) and immune receptor peptidoglycan-recognition protein LC (PGRP-LC, Cflo_N_g10272). As an important component of ubiquitin-proteasome pathway SkpA is involved in Immune Deficiency (IMD) pathway regulation in *D. melanogaster*^30^. Since PGRP-LC is also a regulator of the ant IMD pathway^3^, the interaction we identified suggests that SkpA can modulate the IMD pathway by the interaction with PGRP-LC. Not only the interaction between protein complexes such as laminin subunit beta-1 (Cflo_N_g14102) and laminin subunit gamma-1 (Cflo_N_g9869) but also the interaction between Cflo_N_g14102 and C-type lectin precursor (Cflo_N_g765) was resolved (see Datasheets 3 in Supplementary Material for all the interactions).

**Figure 2:**
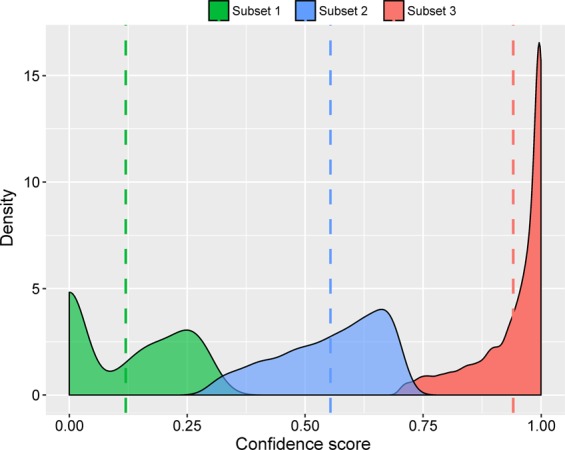
Density plot of the confidence scores for interactions in the *C. floridanus* interactome. How sure are the predicted protein-protein interactions? The distribution of the different confidence levels were computed with CAPPIC^31^. Score distributions were separated into low, medium and high confidence category and the density for each category was plotted. In the three subsets scores range between 0 and 0.3 for subset 1 (green, low confidence), 0.3 and 0.7 for subset 2 (blue, medium confidence) and 0.7 and 1 for subset 3 (red, high confidence). Stippled coloured lines indicate median values for the different categories.

To further supplement the proposed ant interactome, we performed a topology-based scoring of the network. The method CAPPIC^31^ used the intrinsic modularity of PPI network for assessing the confidence of individual interactions. 88.5% of the total interactions are high confidence (Fig. 2) while 9.65% were assigned to medium confidence and 1.8% to low confidence.

We applied the Mann-Whitney test to compare the average confidence scores of all four PPI networks and observed significant increase of confidence score for the first three steps from the preliminary network through DDI mediated filtering and localization-based filtering (Supplementary Fig. 1). The mean confidence score of the final interactome, after the isoform merging, did not change much. This is because the merging of this last step also eliminated some high confidence PPIs mediated by the isoforms. Nevertheless, the comparison of the proportions of high-confidence PPIs in the preliminary interactome and the final ant interactome indicates that it has a significantly increased number of high confidence interactions (in the preliminary network these are 78%, in the final 89%; Fisher’s exact test p-value < 2.2e-16). Note that the applied filtering steps also eliminated most of the low confidence PPIs (see low confidence zone in Supplementary Fig. 1). To further confirm the elimination after successive filtering steps, we compared the low confidence PPIs proportions in all four interactomes in a pairwise way with Fisher’s exact test and show a significant decrease in the number of the low confidence PPIs between the preliminary, DDI-filtered and localization-filtered interactomes (in preliminary 4.5%, in DDI-filtered 2,2%, in localization-filtered 1.6% with maximum p-value < 3.4e-05). These analyses clearly demonstrate the improvement of network quality after filtering steps.

### Network analysis of *C. floridanus* interactome and accuracy assessment

The resulting PPI summarizes the whole network and reveals central connecting nodes. The final high confidence ant interactome showed a clustering coefficient of 0.094 with a mean shortest path length of 4.359, network diameter 14 and an average degree of 6.970. As a typical biological network^32–34^ it shows small-world connectivity and scale-free topology.

We further tested whether the proposed interactome aligns with the properties of a real biological network. To assess this, we derived three independent datasets and compared their topological properties with the proposed network. The average z-statistic value (Datasheet 5 in Supplementary Material) clearly indicates comparatively less variation of the ant interactome from the ‘Barabási-Albert scale free model’ (z-statistic = 23.06, −5.28) in terms of clustering coefficient and mean shortest path. However, the differences were high while comparing that of with ‘Watts–Strogatz small world graph model’ (z-statistic = −30.95, −58.49) and ‘Erdős-Rényi flat-random’ network model (z-statistic = 171.03, −52.72). Scale-free networks have been often observed in biological systems such as PPI and gene regulatory networks^35^, therefore the bias towards such a network is an indicator of the equality of the reconstructed ant interactome. To test another factor, the degree distribution of the ant interactome was much closer to Watts–Strogatz model (z-statistic = 0.49), although the differences with Barabási-Albert model was not too high (z-statistic = 2.45). The nodes in the network obey a power-law distribution indicating a typical, biological small-world and scale-free network.

### Gene ontology (GO) enrichment analysis

The molecular function GO term over-representation analysis indicates enriched protein functions in the ant networks (FDR <0.05; Table 2 and Datasheet 6 in Supplementary Material). Over-represented functional categories include the term ‘binding’ as to be expected from the PPI construction and a validation criterion. Out of 2804 proteins annotated as GO term GO:0005488 ‘binding’ in *C. floridanus* proteome, 46.11% proteins are present in the final interactome. In total, 64 binding-related GO terms were identified constituting 34.97% of all over-represented GO terms. We only found the under-representation of two GO terms: GO:0003964 ‘RNA-directed DNA polymerase activity’ and GO:0034061 ‘DNA polymerase activity’. This indicates during the filtering we did not lose most of the functional proteins that are involved in molecular binding.

**Table 2:** Top 20 over-represented GO molecular function terms in the ant-interactome^a^.

GO ID	GO molecular function term	FDR
GO:0005488	Binding	3.27E-130
GO:0003824	Catalytic activity	8.17E-92
GO:0097159	Organic cyclic compound binding	9.44E-88
GO:1901363	Heterocyclic compound binding	9.44E-88
GO:0036094	Small molecule binding	3.59E-80
GO:1901265	Nucleoside phosphate binding	7.83E-78
GO:0000166	Nucleotide binding	7.83E-78
GO:0043168	Anion binding	2.45E-66
GO:0017076	Purine nucleotide binding	5.15E-59
GO:0032553	Ribonucleotide binding	7.09E-59
GO:0032555	Purine ribonucleotide binding	1.76E-58
GO:0035639	Purine ribonucleoside triphosphate binding	2.85E-58
GO:0097367	Carbohydrate derivative binding	2.68E-55
GO:0043167	Ion binding	3.31E-52
GO:0016817	Hydrolase activity, acting on acid anhydrides	2.18E-39
GO:0005515	Protein binding	2.18E-39
GO:0016818	Hydrolase activity, acting on acid anhydrides, in phosphorus-containing anhydrides	9.29E-39
GO:0016462	Pyrophosphatase activity	6.95E-38
GO:0017111	Nucleoside-triphosphatase activity	3.90E-37
GO:0030554	Adenyl nucleotide binding	3.90E-37

^a^See Datasheet 6 in Supplementary material for the complete list of over-represented GO molecular functions terms in ant-interactome.

**Figure 3:**
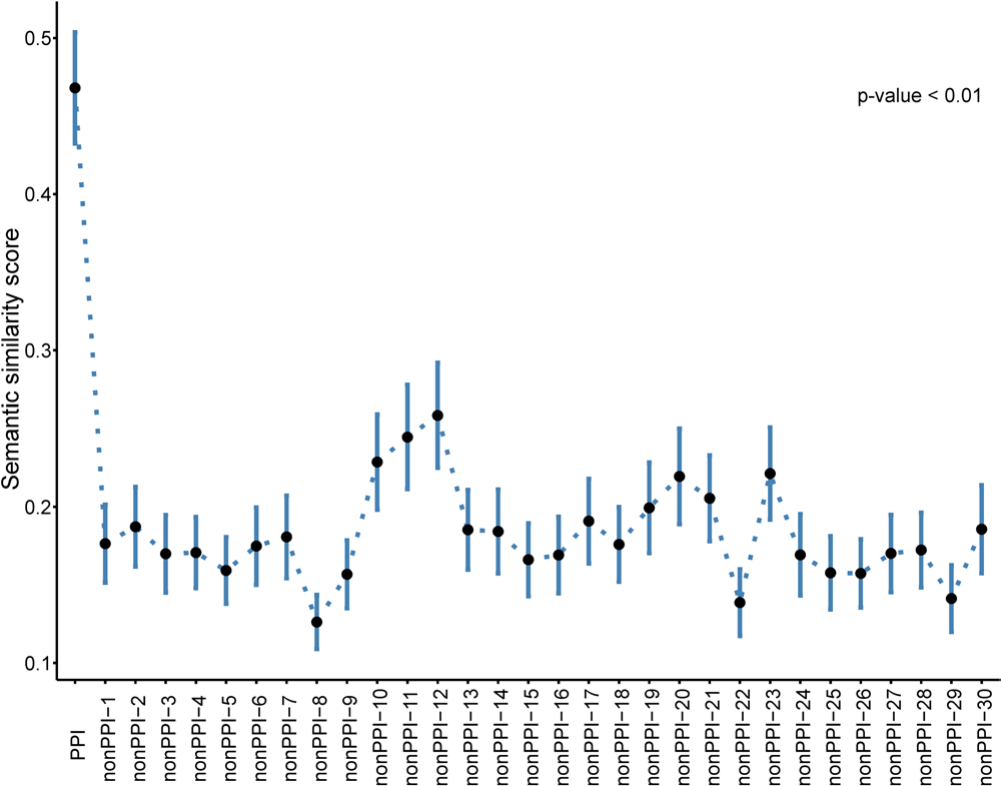
Pairwise Gene Ontology (GO) similarity of the ant interactome compared against non-interacting proteins. The high average similarity score of the ant interactome (PPI, left) stands out against the low similarity scores of the non-interacting PPIs. Semantic similarity score between interacting (PPI) and non-interacting (nonPPI) protein pairs were compared in a pairwise fashion using the Mann-Whitney U test. The Average scores for semantic similarity in molecular function level-4 GO annotations of interacting proteins and 30 random networks of non-interacting proteins are shown.

We further compared the semantic similarity scores of the interacting pairs with the random networks of non-interacting proteins. We first assigned the level-4 GO annotations (for molecular function) to all the proteins coded by the ant genome using Blast2GO^36^. Next, we used the GOGO algorithm^37^ to measure the semantic similarity scores of the high confidence interacting pairs in the proposed ant interactome. We further generated 30 random networks each with 100 random interactions among the proteins that were assigned to level-4 molecular function GO annotations using a custom-made Perl script which can be accessed from the GitHub repository (https://github.com/ShishirGupta-Wu/ant_ppi). We made sure the random networks did not contain any proteins pairs apparent in the preliminary interactome. Using the GOGO algorithm^37^ semantic similarity scores were also assigned to the random networks (non-PPIs) and these scores were further compared with the interacting proteins in a pairwise way using the Mann-Whitney U test. We observed that the interacting protein set had not only the highest average score of 0,47, this was also well separated and significantly higher than the average score in all the 30 non-PPI sets (Fig. 3). This comparison demonstrates the interactions in our calculated ant interactome are functionally relevant and clearly different from random networks.

### *C. floridanus* interactome protein conservation compared with seven organisms

**Figure 4:**
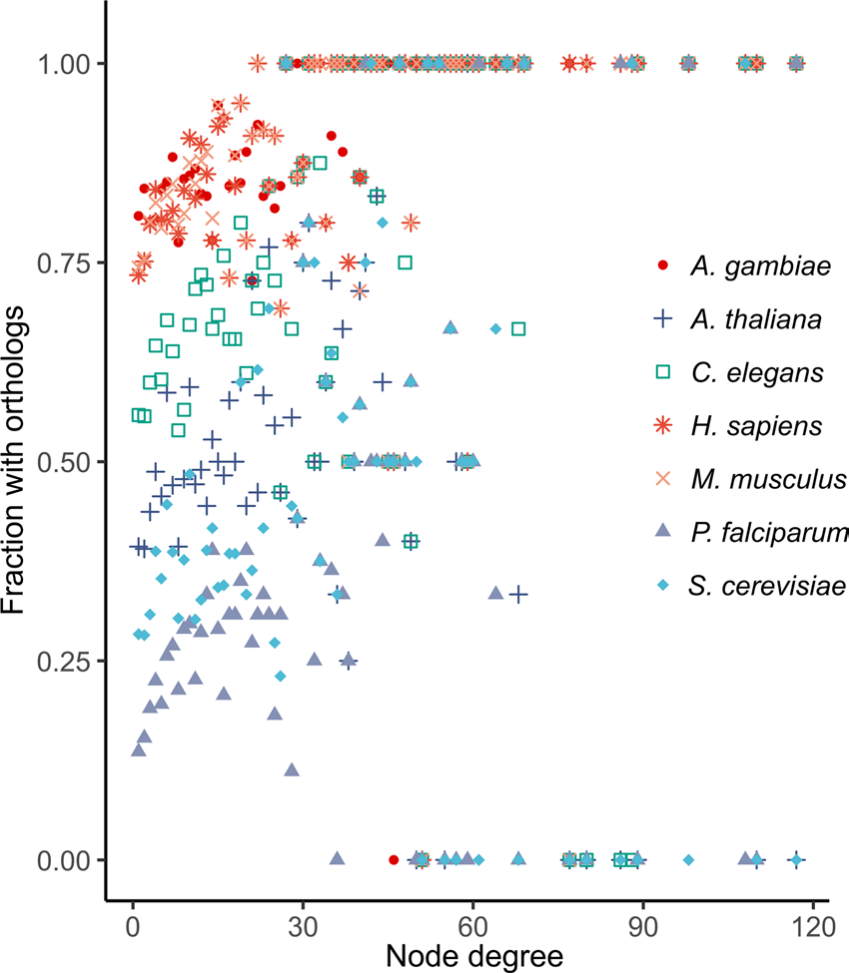
Conservation of interacting proteins by degree in different model species. The conservation level of ant interactome proteins with varying degrees are shown in each analyzed species and in general the interactions are well confirmed by several species (symbols listed on the right, middle). Higher connceted proteins (right) become rare and so if present in the compared model organism, they are fully confirmed (1.00, top) or nothing is found in some other model species (0.00, bottom). Each protein in the *C. floridanus* interactome was examined for orthologous proteins in the seven organisms, and binned according to degree. The proportion of each bin with orthologous proteins in shown. A trendline is not shown on the graph since the data is analyzed with Spearman’s rank correlation and a trendline could be misleading.

Proteins that perform essential functions are expected to be evolutionary conserved. We further investigated the evolutionary conservation of ant interactome proteins. Higher degree proteins are generally evolutionary better conserved^38^, some caveats are discussed in^39,40^. To analyze this, node degree and the fraction of proteins present in the ant interactome that are conserved in different model organisms were compared. It turns out that in general the interactions are conserved and supported by most species tested and not just by one (Fig. 4). For exact quantification we did not check the possible restricted conservation of the binary ant PPIs, but more general the conservation of proteins that are present in the ant interactome and have orthologs in seven other species. For instance, in the ant interactome there are 535 proteins of degree 2. Out of these 535 proteins 451 have an ortholog in Anopheles, 209 have an ortholog in Arabidopsis, 298 have an ortholog in *C. elegans*, 404 have an ortholog in mouse, 82 have an ortholog in plasmodium, 151 have an ortholog in yeast, and 402 have a human ortholog.

There was a positive correlation between degree and conservation in the evolutionary closest analyzed species *A. gambiae* (Spearman’s rank r = 0.62, p-value = 3.5e-09). Similar correlations are observed between ant and human (r = 0.60), and mouse (r = 0.51). Between ant and worm the correlation was weak (r = 0.33), while no significant correlation is observed between ant and *A. thaliana*, *P. falciparum*, and yeast. An ortholog table is provided in Datasheet 7 in Supplementary Material.

### Overall conservation and infection induced hubs and bottlenecks in the ant interactome

**Figure 5:**
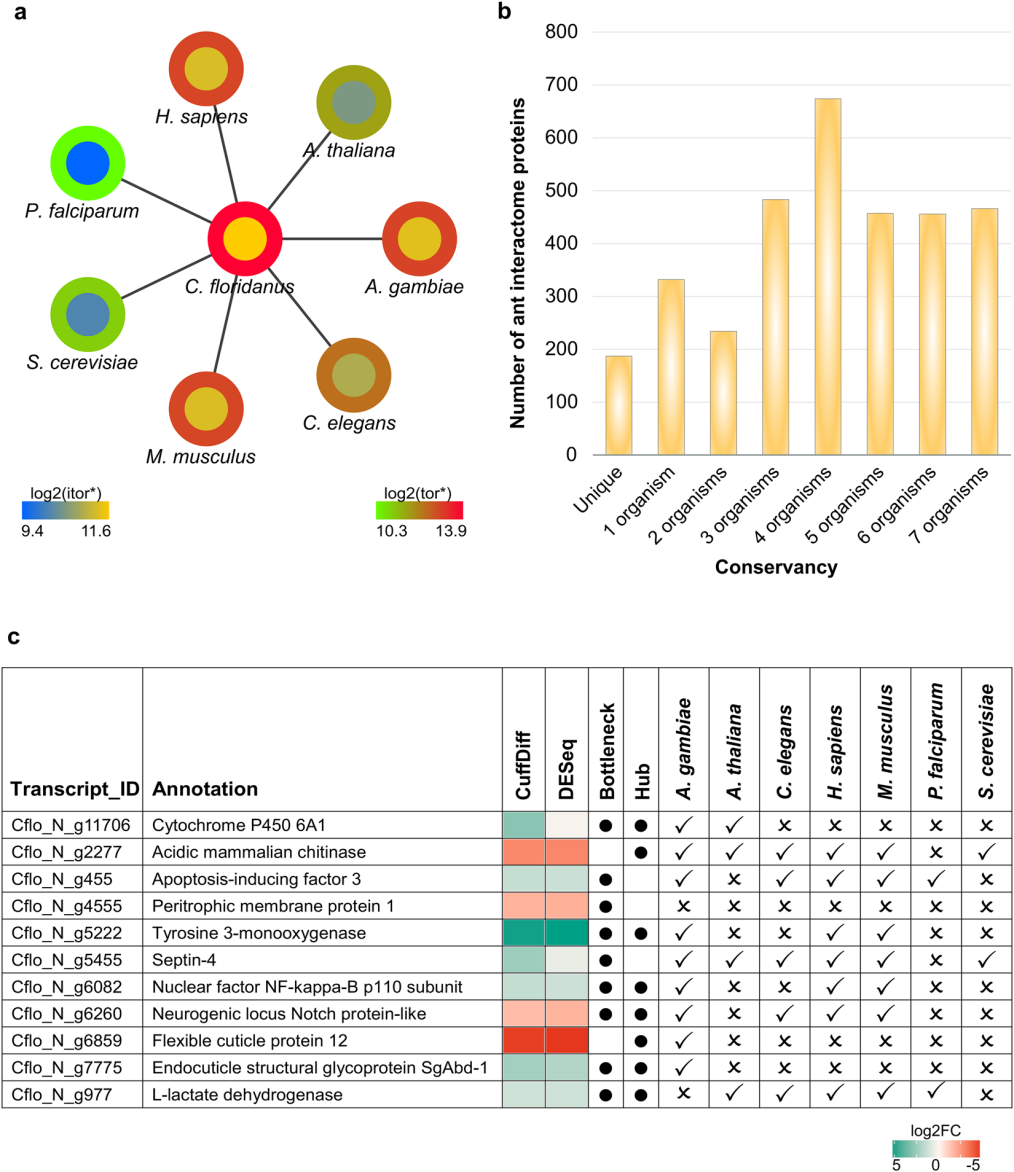
Conservation of *C. floridanus* proteins. (**a**) Network showing the similarity *of C. floridanus* proteins (central node) with other organisms proteins (peripheral nodes) quantitively by color codes. Color gradients represent the number of proteins in each species that have orthologs in either *C. floridanus* interactome (itor, inner circle) or in the whole *C. floridanus* proteome (tor, outer circle). A similar color to the ant node shows close orthology relation while nodes of more distant species have different colors. A correlation between the colors of two circles is expected to show that the interactome successfully represent the orthology relationships between ant and other species. For instance, if both circles of a species show a similar color to ant, it depicts that the close orthology to ant can be also observed in the interactome. Pearson’s Chi-squared test with Yates’ continuity correction showed relationship (p-value < 2.2e-16) between the total ant proteins orthologs and interactome proteins orthologs for each corresponding organisms. (**b**) Conservation of ant interactome proteins in set of any analyzed organisms. Number of ant interactome proteins that are unique to ant and that are orthologous to proteins in different numbers of analyzed species is shown. (**c**) Differentially expressed top hubs and bottlenecks (represented by black dots) and presence/absence of their orthologs in other seven organisms. Differential expression values (log2FC) of hubs and bottlenecks in bacterial infection of *C. floridanus* are represented by a color gradient.

We also evaluated the overall conservation of all the ant proteins with the other seven model organisms and compared the relatedness of the ant interactome proteins using the chi-square test. The analysis indicated the relatedness of corresponding proportions with p-value < 0.05 in each case. The differences in the number of orthologs can be clearly visualized (Fig. 5a) in case of ant comparison with protozoan parasite, yeast and plant.

Due to the large phylogenetic distance to these three organisms there are less orthologs but these are well conserved (chi-square test).

The remaining set of the other four organisms including insect, human, mouse and worm together consists of/contains higher number of orthologs in comparison to the ant proteins (Fig. 5b and Datasheet 7 in Supplementary Material). 187 proteins of the ant interactome are ant-specific in this comparison: they do not have orthologs in any of the analysed organisms (Fig. 5b). The analysis of central topological properties of a PPI network helps to identify key multifunctional components of the network^41^. Infection induced proteins of *C. floridanus* are conserved in related organisms including key interactions. The degree of the node^42^ and the betweenness centrality^43^ represent the most important properties in the PPI network because of their role in maintaining the functional integrity and connectivity of the network. Proteins with high degree are termed as hubs while the proteins with high betweenness centrality are termed bottlenecks.

We applied Fisher’s exact test to compare the proportion of multi-localized proteins in hubs and bottlenecks to non-hubs and non-bottlenecks, respectively. Supplementary Fig. 2 shows differences between the localization of bottlenecks and hubs of the ant interactome. For bottleneck proteins, 70% were found to be multi-localized (versus 56% for non-bottleneck proteins; significant difference; p = 9.6 × 10^−10^). On the other hand, 62% of the hub proteins had multiple localizations (versus 56% for non-hub proteins; significant difference, p = 0.001575).

Integration of the RNASeq data^3^ with the ant interactome revealed differentially expressed infection-induced hubs and bottlenecks during the bacterial infection of *C. floridanus* (Fig. 5c). These include also well-known key proteins involved in *C. floridanus* immune response such as nuclear factor NF-kappa-B p110 (Relish, Cflo_N_g6082), acidic mammalian chitinase (Cflo_N_g2277), as well as stress-related protein cytochromes P450 6A1 (Cflo_N_g11706)^3^. Given the high importance of hubs and bottlenecks in PPI networks and their differential expression during bacterial infection, all the identified proteins are expected to participate in the defense against bacterial pathogen, and hence can also be examined for decoding immune mechanisms. The insect peritrophic membrane (PM) imposes protective physical barriers over the midgut epithelium^44^. The PM related proteins have shown their potential as targets for pest control^45,46^. Therefore, the important ant peritrophic membrane protein 1 (Cflo_N_g4555) (Fig. 5c) with no human homology could be further tested as a potential pest target. However, differential expression does not guarantee a protein to be the best target^47,48^ and therefore, other topologically important proteins in the network without human homology (Datasheet 7 in Supplementary Material) should also be considered as potential pest targets in future.

## Conclusions

Our curated ant interactome is the first large-scale PPI network of an ant. It allows besides numerous analysis of network biology to study how different cellular processes connect to each other including hub proteins and different types of crosstalk, for instance in immunity.

Similarly, the PPI maps of other sequenced ants can be reliably predicted using the interologs of the reconstructed high-confidence *C. floridanus* interactome. Moreover, detailed cross-validation, comparison with random networks, GO annotation, and conservation analysis support the high quality of the resulting ant interactome and its construction steps. The network analysis including evolutionary conserved network proteins further suggest that topologically important proteins could also be exploited as future pest targets. For instance, cytochrome P450 6A1 (Cflo_N_g11706), peritrophic membrane protein 1 (Cflo_N_g4555), flexible cuticle protein 12 (Cflo_N_g6859), endocuticle structural glycoprotein SgAbd-1 (Cflo_N_g7775) were identified as topologically important differentially expressed proteins with no human orthologs. Nevertheless, specific interactions highlighted from our global analysis will need individual follow up by detailed investigations.

## Materials and Methods

### Reconstructing protein-protein interaction map of *C. floridanus*

We compiled the list of experimentally verified high-confidence PPIs available in Database of interacting proteins (DIP)^49^, *D. melanogaster* PPIs from DroID^50^ database which includes data from different studies including interactions from high throughput Gal4 proteome-wide yeast two-hybrid (Y2H) screens^32^, LexA Y2H system screens^51–53^, PPIs from fly protein interaction map^54^, interactions determined in large-scale co-affinity purification (co-AP)/MS screens^55,56^, interactions from BIND^57^, BioGRID^58^, MINT^59^, IntAct^60^, and databases available in DroID v2014_10.

The *C. floridanus* interologs of the entire template PPIs were determined using orthology predictions from the software InParanoid^20,61^ and OrthoMCL^21^. These were further customized using own perl and bash scripts. For DIP interactors we used the default parameters of InParanoid. For the fly data orthology was determined using the stricter Blosum80 matrix. For the OrthoMCL based interologs mapping a Blast e-value of 1e-05 was used and the MCL inflation index set to 1.5. InParanoid distinguished seed orthologs with co-orthologs and left fewer possibilities of mixing outparalogs in orthologous clusters. Consensus predictions of InParanoid and OrthoMCL were added to InParanoid seed orthologs to create a set of interologs.

### Pruning PPIs with domain-domain interactions

The amino acid sequences of non-redundant preliminary PPIs were extracted and domains were assigned to them using Pfam version 27.0^62^. The list of non-redundant domain-domain interactions was prepared from the meta-databases Domine^63^, DIMA 3.0^64^ and IDDI database^65^. These use complexes available in the Protein Data Bank (PDB)^66^ to identify by interacting domains the Pfam families containing these domains. These Pfam families are then predicted to be interacting. This list was used to parse the template PPIs. All interactions were categorized whether they are supported (good interactions, used for further filtering steps) or not by domain-domain interactions (DDIs).

### Subcellular localization filtering

The subcellular localization of *C. floridanus* proteins was determined with orthology to Swiss-Prot proteins and the extended version of KnowPredsite^67^ available at UniLoc server (bioapp.iis.sinica.edu.tw/UniLoc/), a knowledge-based classifier for protein subcellular localization. If in a binary interaction, both proteins do not share the same localization or at least one compartment in multiple localized proteins, the interaction was ruled out as probable not occurring.

### Isoform filtering

The information on *C. floridanus* protein isoforms and their function was extracted from our previous publication of *C. floridanus* re-annotation and transcriptome sequencing^3,68^. To reduce network complexity and noise, isoforms of any specific protein present in the network were represented as a single node. Although, the data files for all the networks are provided in the Supplementary Tables (1–5) which allow interested readers to analyze the network of their choice further if they wish.

### Assigning the confidence score

In fact, the preliminary network is filtered successively as mentioned above to reconstruct the final network, in this way the final network is already of high-confidence as many network biologists working on PPI networks have used DDIs and subcellular localization either to increase confidence or validate the interacting pairs. Here additionally we used topology-based method CAPPIC (cluster-based assessment of protein-protein interaction confidence) to assign the interaction confidence score^31,69^ in the filtered network. In brief, CAPPIC calculations are based on the assumption that the proteins existing in the same network module are expected to have a higher number of common neighbours (neighbourhood interconnectedness^70^), and a short path length inbetween^71^. For scoring the confidence level, CAPPIC first performs the clustering of the network using a robust clustering algorithm, Markov Cluster (MCL)^72^ and then scores the interactions according to their level of compliance with the basic assumptions of topology-based methods. For the clustering we used an MCL inflation value of 1.5. Scores were classified to three subsets; low confidence score between 0 to 0.3, medium confidence score between 0.3 to 0.7, and high confidence score between 0.7 to 1.

### Network analysis and visualization

The *C. floridanus* interactome was subjected to topological analysis using Network Analyzer plugin version 2.7 of Cytoscape version 2.8.1^73^. The node degree distribution, mean path length, network diameter and betweenness centrality (BC) were determined with graph theoretic analysis implemented with CentiScaPe^74^. For the network G(V,E), the BC of node n is defined as follows

1 $$BC(n)=\sum _{s\ne n\ne t}(\frac{{\sigma }_{st}({\rm{n}})}{{\sigma }_{st}}\,)$$

here *s* and *t* are network nodes different from node *n*, *σ*_*st*_ is the number of shortest paths from *s* to *t*, and *σ*_*st*_ (*n*) gives the number of shortest paths from *s* to *t* that goes through node *n*.

Hubs and bottlenecks in the network were identified with cytoHubba^75^. Hubs were defined as proteins connecting with ≥5 proteins. Moreover, top 20% of bottlenecks and hubs were considered for mapping of the RNASeq expression data which was collected from our previous publication^3^.

### Random networks

We generated random networks following the Erdős-Rényi Model^76^, Barabási-Albert Model^77^ and randomized the proposed (final) ant interactome while preserving the total number of interactome nodes using the Network Randomizer plugin^78^ of Cytoscape^73^. A total of 1000 random simulation were employed to generate the undirected random graphs. For all three network sets we computed topological parameters, mean shortest path, degree distribution and clustering coefficient and compared their differences to the native ant interactome using the statistical Z-test^79^.

### Functional annotation

Blast2GO^36^ was used to annotate the Gene Ontology (GO) terms of proteins involved in the reconstructed interactome. Over-representation analyses of GO terms was performed using the Gossip package^80^ of the Blast2GO suite. A two-tailed Fisher’s exact test followed by false discovery rate (FDR) correction for multiple testing^81^ was applied to see the functional difference of ant interactome proteins annotations (foreground set) and full *C. floridanus* proteome annotations^3^ (background set). Only differences having an adjusted p‐value < 0.05 were considered significant.

### Orthology analysis

InParanoid^20^ was used to identify the orthologs of topologically important nodes in seven model organisms: *Anopheles gambiae*, *Arabidopsis thaliana*, *Caenorhabditis elegans*, *Homo sapiens*, *Mus musculus*, *Plasmodium falciparum*, and *Saccharomyces cerevisiae*. Only the ortholog with 100% bootstrap support was considered as true ortholog. As a note of caution, the conservation was calculated rather conservatively demanding double orthology relations. Hence, the absence of an ortholog (Suppl. Datasheet 7) only indicates that the highly restrictive threshold was not met. Generally, a sequence related protein may still be found by less restrictive algorithms (e.g. BLAST).

For exact quantification of the degree of conservation of ant PPIs we did not check the possible restricted conservation of the binary ant PPIs, but more general the conservation of proteins that are present in the ant interactome and have orthologs in seven other species. After calculation of the orthology relationships between ant and other organisms we identified for every degree the occurrence value of the ant interactome and how many orthologs are present in other species. For each organism the fraction of proteins at a particular ant interactome degree is considered as the number of ant protein orthologs at that particular degree and greater divided by the number of proteins in the set.

## Supplementary information

Dataset 1 - 7

Supplementary Information

## Data Availability

All data generated and analysed during this study are included in this published article (and its supplementary Information files). The dataset and codes for random network generation are also available at https://github.com/ShishirGupta-Wu/ant_ppi.
