# Supplementary material for: Genome-wide inference of the Camponotus floridanus protein-protein interaction network using homologous mapping and interacting domain profile pairs
Source: Sci Rep. 2020 Feb 11;10:2334. doi: 10.1038/s41598-020-59344-1 (PMC7012867; doi:10.1038/s41598-020-59344-1)
Supplement: Supplementary file 2 — Supplementary Information [file 41598_2020_59344_MOESM2_ESM.docx]

Genome-wide inference of the *Camponotus floridanus* protein-protein interaction network using homologous mapping and interacting domain profile pairs

Shishir K Gupta^ab^+, Mugdha Srivastava^a^+, Özge Osmanoglu^a^, Thomas Dandekar^a,c,^*

1. Functional Genomics and Systems Biology Group, Department of Bioinformatics, Biocenter, Am Hubland, D-97074 Würzburg, Germany.
2. Department of Microbiology, Biocenter, Am Hubland, D-97074 Würzburg, Germany.
3. EMBL Heidelberg, BioComputing Unit, Meyerhofstraße 1, 69117 Heidelberg, Germany.

**Supplemental Material: Further Data Analysis**

Here we comment further on the analysis flow and different tests and imbed the supplementary figures with figure legends so that their results also are easier to understand.

We applied the Mann-Whitney U test to compare the average confidence scores of all four PPI networks and observed significant increase of confidence score from the preliminary network through DDI mediated filtering and localization-based filtering (Supplementary Fig. 1). However, we did not see the increase in the mean confidence score of final interactome after isoform merging. This is because the merging also eliminates the high confidence PPIs mediated by the isoforms. Although, the comparison of the proportions of high-confidence PPIs in the preliminary interactome and the final ant interactome indicated that the filtered network has significantly increased number of high confidence interactions (in preliminary 78%, in final 89%; Fisher’s exact test p-value < 2.2e-16). Note that the applied filtering steps also eliminated most of the low confidence PPIs (see low confidence zone in Supplementary Fig. 1). To confirm the elimination after successive filtering steps, we compared the low confidence PPIs proportion in all four interactomes in a pairwise way with Fisher’s exact test and observed a significant decrease in number of the low confidence PPIs between the preliminary, DDI-filtered and localization-filtered interactomes (in preliminary 4.5%, in DDI-filtered 2.2%, in localization-filtered 1.6% with maximum p-value < 3.4e-05). These analyses clearly indicate the improvement of network quality after the filtering steps (**Supplementary Fig. 1)**


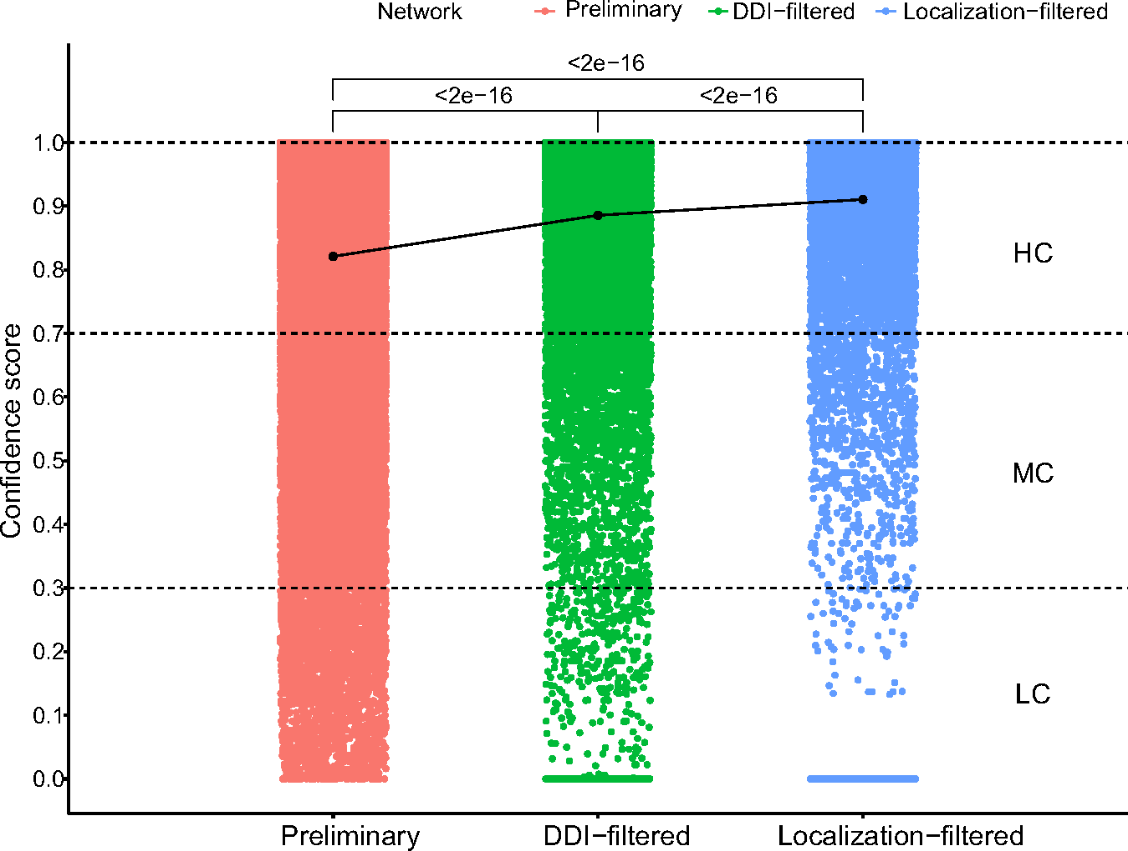


***Supplementary Fig. 1.*** ***Network quality improvement.*** *Effect of filtering steps observed by the change in average confidence score in successive interactomes. Confidence intervals shown with dashed lines. Decrease in the amount of low-confidence interactions can be observed under the 0.3 line. Increase in mean after each filtering step can be observed. HC, MC and LC represent high confidence, medium confidence and low confidence zones respectively.*

The mean confidence score of the final interactome, after the isoform merging, did not change much. This is because the merging of this last step also eliminated some high confidence PPIs mediated by the isoforms. Nevertheless, the comparison of the proportions of high-confidence PPIs in the preliminary interactome and the final ant interactome indicates that it has a significantly increased number of high confidence interactions (in the preliminary network these are 78%, in the final 89%; Fisher’s exact test p-value < 2.2e-16). Note that the applied filtering steps also eliminated most of the low confidence PPIs (see low confidence zone in Supplementary Fig. 1).

**Pairwise GO similarities:**

To evaluate the functional relevance of PPIs in the final ant interactome we compared the semantic similarity scores of the interacting pairs with the random networks of non-interacting proteins. We first assigned the level-4 GO annotations (for molecular function) to all the proteins coded by the ant genome using Blast2GO.


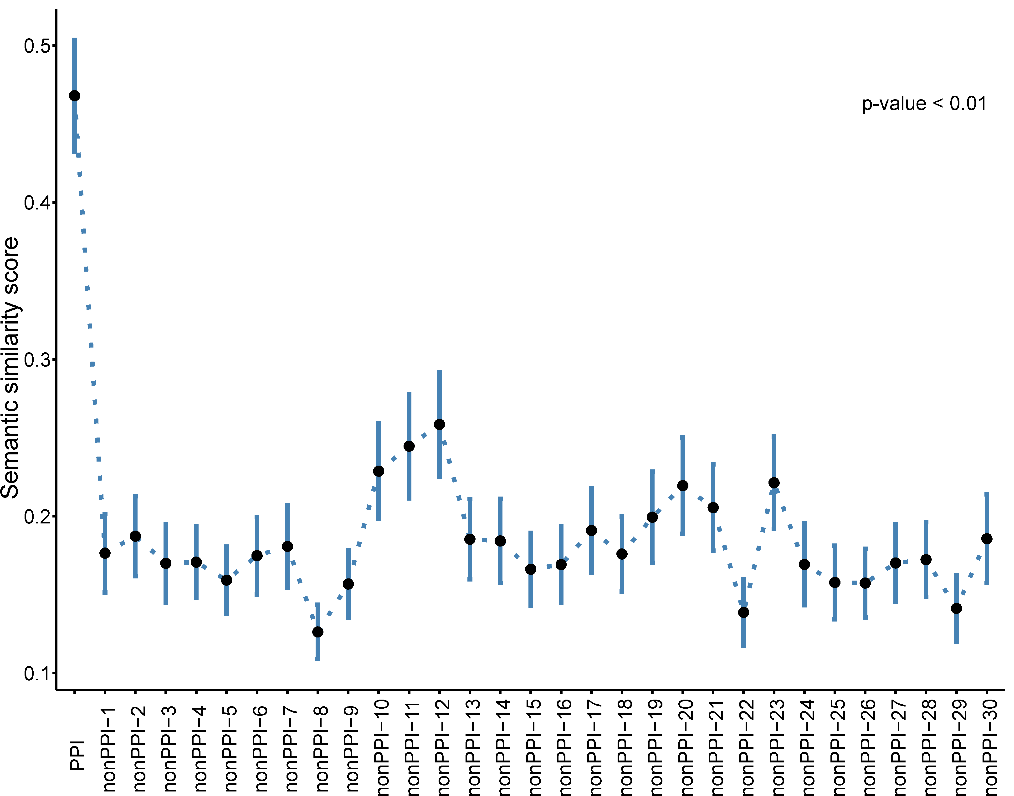


***Figure 3.*** ***Pairwise Gene Ontology (GO) similarity comparison.*** *Semantic similarity score between interacting (PPI) and non-interacting (nonPPI) protein pairs were compared in a pairwise fashion using the Mann-Whitney U test. Average scores for semantic similarity in molecular function level-4 GO annotations of interacting proteins and 30 random networks of non-interacting proteins are shown.*

Next, we used the recently published GOGO algorithm (<https://www.nature.com/articles/s41598-018-33219-y>) (Zhao and Wang 2018) to measure the semantic similarity score of the high-confidence interacting pairs in the proposed ant interactome. We further generated the 30 random networks each with 100 random interactions among the proteins that were assigned to level-4 molecular function GO annotations using custom Perl script which can be accessed from the GitHub repository (<https://github.com/ShishirGupta-Wu/ant_ppi>). We made sure the random network did not contain any proteins pairs that appeared in the preliminary interactome. Using GOGO algorithm semantic similarity scores were assigned to the random networks (non-PPIs) and further these scores were compared with the interacting proteins in a pairwise way using Mann-Whitney U test. We observed that interacting protein set had the highest average score of 0,47 and was significantly higher than the average score in all the 30 non-PPI set (Figure 3 in results, shown above again).

**Differences between proteins localized in single *versus* multiple subcellular compartments**

We applied Fisher’s exact test to compare the proportion of multi-localized proteins in hubs and bottlenecks to non-hubs and non-bottlenecks, respectively. Supplementary Fig. 2 shows the systematic differences between the localization of bottlenecks and hubs of the ant interactome. For bottleneck proteins, 70% was found to be multi-localized, while only 56% of non-bottlenecks had multiple subcellular localizations (p-value = 9.6e-10). On the other hand, 62% of hubs had multiple localization, whereas the proportion for non-hubs was 56% (p-value = 0.001575).


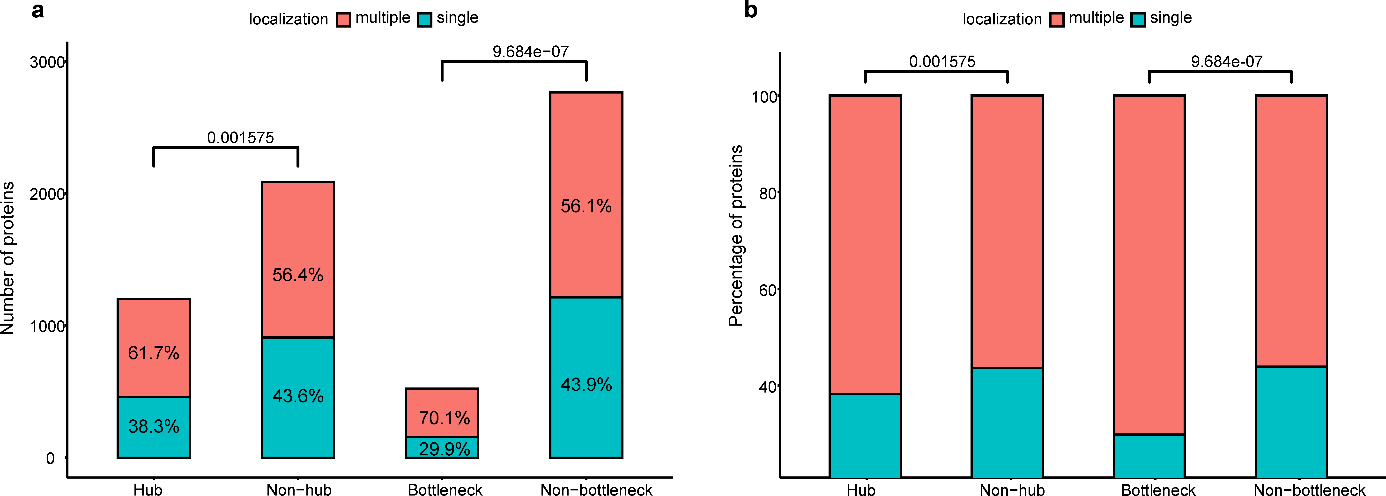


***Supplementary Fig. 2.*** ***Localization of hubs and bottlenecks.*** *(a) Protein sets of hubs and bottlenecks tend to have a higher proportion of multi-localized proteins than ones of non-hubs and non-bottlenecks, respectively (61.7% of hubs vs 56.4% of non-hubs, 70.1% of bottlenecks vs. 56.1% of non-bottlenecks show multiple localization). (b) Similar plot is shown with normalized bar length to 100%.*

**Further References for our analyses methods**

Conesa A, et al. 2005. Blast2GO: a universal tool for annotation, visualization and analysis in functional genomics research. Bioinformatics 21: 3674-3676. doi: 10.1093/bioinformatics/bti610

Goldberg DS, Roth FP 2003. Assessing experimentally derived interactions in a small world. Proc Natl Acad Sci U S A 100: 4372-4376. doi: 10.1073/pnas.0735871100

Kuchaiev O, Rasajski M, Higham DJ, Przulj N 2009. Geometric de-noising of protein-protein interaction networks. PLoS Comput Biol 5: e1000454. doi: 10.1371/journal.pcbi.1000454

Vlasblom J, Wodak SJ 2009. Markov clustering versus affinity propagation for the partitioning of protein interaction graphs. BMC Bioinformatics 10: 99. doi: 10.1186/1471-2105-10-99

Zhao C, Wang Z 2018. GOGO: An improved algorithm to measure the semantic similarity between gene ontology terms. Sci Rep 8: 15107. doi: 10.1038/s41598-018-33219-y
